# Penetrating insights?

**DOI:** 10.3389/fmicb.2012.00382

**Published:** 2012-10-31

**Authors:** Dave Speijer

**Affiliations:** Department of Medical Biochemistry, Academic Medical Center, University of AmsterdamAmsterdam, Netherlands

**A commentary on**

**Non-biased enrichment does not improve quantitative proteomic delineation of reovirus T3D-infected HeLa cell protein alterations**

by Jiang, J., Opanubi, K. J., and Coombs, K. M. (2012). Front. Microbio. 3:310. doi: 10.3389/fmicb.2012.00310

In this special Frontiers in Microbiology issue focusing on “Global host proteomic responses to virus infection,” Coombs and colleagues (Jiang et al., 2012) make a valiant effort to look deeper into (changes in) the proteome of HeLa cells upon reovirus infection. Using some of the latest proteomic techniques, they present a high quality analysis of quantified alterations in the protein content of HeLa cells with, or without, infection by reovirus T3D. The study of the relatively harmless mammalian reoviruses received a boost from their potential to selectively kill certain types of cancer cells (Coffey et al., 1998). Proteomic studies, comparing all proteins and their post translational modifications in a quantitative fashion between control and reovirus infected cells therefore became crucial. However, all proteomic studies are hampered by the large differences in natural protein concentrations. In mass spectrometric (ms) practice, the most conspicuous protein restricts the level of detection to proteins that are, at most, about a thousand fold less abundant. Methods used to overcome this limitation either purge the most abundant, or specifically purify the less abundant proteins. Of course such biased approaches have rather big drawbacks themselves, related to the method of purging and the choice of purification procedure. “Unbiased” approaches, leveling the playing field without prior loss of possibly interesting proteins or enrichment based on known interactions, come in two flavors. One of them is based on the use of antibodies against “every” protein made by a certain organism as illustrated by human tissue profiling (Uhlen and Ponten, 2005). This is a non-ms technique, although immune precipitations can be combined with further ms analysis. The technique would indeed allow sensitive detection of very low abundant proteins, but relies on getting an effective epitope for every gene product and is rather costly. It also has to focus on the most common protein form and will be difficult to use for the detection of less abundant forms, let alone post translational modifications.

The only really unbiased approach available seems to be the use of hexapeptide combinatorial ligand libraries to enrich for all low abundant proteins (Boschetti and Righetti, 2008). The basic idea is simple and rather elegant: generate “random” peptide libraries, coat individual beads with single peptide forms, and allow your protein sample to interact with complex beads mixtures. Highly abundant proteins will saturate the beads they interact with quickly, and the large unbound majority will be washed away. However, proteins that are much less abundant will also find peptide targets to their liking and be retained. To give a somewhat more precise description: the abundance advantage of certain proteins (differences in mol L^−1^) has to be *more* than compensated for by differing binding affinities (again in mol L^−1^). Prior work showed hexapeptide libraries (with a combinatorial complexity of 6.4 × 10^7^ peptides) to be optimal in this respect. Of course, not all amino acids in the hexamers contribute to the (strength of) interaction with proteins in the sample equally, three hydrophobic ones (Phe, Trp, and Tyr) and the three basic ones (Arg, His, and Lys) presumably being the most important. This (ProteoMiner) technique is the one that Jiang et al. decided to use. However, in their abstract it is concluded that: “Comparisons of the *r*^2^ correlations, degree of dataset overlap, and numbers of peptides detected suggest that non-biased enrichment approaches may not provide additional data to allow deeper quantitative and comparative mining of complex proteomes.” It is refreshing that these authors highlight what could be seen as a “negative” finding. In presenting evidence suggesting that in *this case* non-biased enrichment strategies do not seem to allow us to delve much deeper into the proteome, the authors give us the latest installment in an ongoing saga regarding the effectivity of using a ProteoMiner approach to get a peek at low abundant proteins in an unbiased fashion. The approach has already been used extensively in one of the most important, but at the same time most challenging, areas of clinical research: biomarker discovery in serum (which has tremendous differences in protein abundancies at ~10^10^ differences in concentration). Discussions regarding the effectivity of using hexamers seem to have been resolved in favor of the approach, although peptide elution had to be performed at three separate pH values to get full peptide diversity (Bandow, 2010; Di Girolamo et al., 2011). In regards to cellular extracts, an extreme example of protein “imbalance” is found in erythrocytes with 98% hemoglobin. The 2% that remains was much more easily explored upon the use of two different hexamer peptide sets (Roux-Dalvai et al., 2008). Even more striking are the results obtained by Yates and colleagues using “normal” Hela cells: they obtain improvements in silverstained 2D gels as well as statistically significant differences in proteome sets using ms analysis of fractions with or without prior ProteoMiner beads incubation (Fonslow et al., 2011).

So what is going on in the case of these reovirus infected HeLa cells? Actually, the conflicting data can presumably be resolved by highlighting the following two points:

1. As in the case of the earlier discussion, *the elution method seems to be crucial*. The ProteoMiner elution buffer used by the Yates group for the 2D silverstained gels is identical to the one used by Jiang et al., *but they digest their proteins on the beads upon 8 M urea denaturation* for further ms analysis (Fonslow et al., 2011). For the differences in 2D analysis followed by silverstaining, the normal protocol seems to be sufficient, but for efficient mass analysis more harsh conditions are clearly needed. For more important insights regarding the use of ProteoMiner beads as a powerful method of proteome equalization see Righetti et al. (2012).
2. Based on the fact that comparisons of *r*^2^ correlations, dataset overlaps, and numbers of peptides detected were comparable to those found with biological replicates the authors were correct in stating that the enrichment approach did not provide a significant deeper quantitative mining of the proteome under study. However, looking at the Venn diagram of shared and specific proteins found in different experiments (Jiang et al., Figure 3A), I am convinced that future analysis of the “extra” groups of proteins found with the hexameric non-biased enrichment approaches in the nuclear and cytoplasmic fractions will show them to contain more low abundant proteins than the controls.

Indeed, future experiments to check for improvement in detection of low abundant proteins should again be performed with virus infected cells. These systems are especially suited for such an analysis as they contain a specific set of viral proteins that are completely absent from control cells, thus functioning as ideal positive controls. Not only that, many viruses have a pronounced dynamic range of viral protein (form)s of their own, making the challenge of a correct quantitative proteomic description even bigger. Despite not always living up to their full potential yet, random hexapeptide libraries seem to be on track to level the proteomic playing field in the near future.
